# PD-L1/PD-1 crosstalk in colorectal cancer: are we targeting the right cells?

**DOI:** 10.1186/s12885-018-4853-0

**Published:** 2018-10-03

**Authors:** Ramón Cantero-Cid, José Casas-Martin, Enrique Hernández-Jiménez, Carolina Cubillos-Zapata, Aníbal Varela-Serrano, José Avendaño-Ortiz, Marta Casarrubios, Karla Montalbán-Hernández, Ignacio Villacañas-Gil, Laura Guerra-Pastrián, Begoña Peinado, Cristóbal Marcano, Luis A Aguirre, Eduardo López-Collazo

**Affiliations:** 1 The Innate Immune Response Group, IdiPAZ, La Paz University Hospital, Madrid, Spain; 2 Tumour Immunology Laboratory, IdiPAZ, Madrid, Spain; 3 Surgery Department, La Paz University Hospital, Madrid, Spain; 4 Centre for Biomedical Research Network, CIBEres, Madrid, Spain; 5 Pathologic Anatomy Service, La Paz University Hospital, Madrid, Spain

**Keywords:** Colorectal cancer, Immune checkpoints, MMR status, PD-L1/PD-1, T-cell exhaustion

## Abstract

**Background:**

The analysis of tumour-infiltrating immune cells within patients’ tumour samples in colorectal cancer (CRC) has become an independent predictor of patient survival. The tumour microenvironment and the immune checkpoints, such as PD-L1/PD-1, are relevant to the prognoses and also appear to be relevant for further CRC therapies.

**Methods:**

We analysed the presence and features of the infiltrated monocyte/macrophage and lymphocyte populations in both tumour and peritumour samples from patients with CRC (*n* = 15).

**Results:**

We detected a large number of CD14^+^ monocytes/macrophages with an alternative phenotype (CD64^+^CD163^+^) and CD4^+^ lymphocytes that infiltrated the tumour, but not the peritumour area. The monocytes/macrophages expressed PD-L1, whereas the lymphocytes were PD-1^+^; however, we did not find high PD-L1 levels in the tumour cells. Coculture of circulating naïve human monocytes/macrophages and lymphocytes with tumour cells from patients with proficient mismatch repair CRC induced both an alternative phenotype with higher expression of PD-L1 in CD14^+^ cells and the T-cell exhaustion phenomenon. The addition of an α-PD-1 antibody restored lymphocyte proliferation.

**Conclusion:**

These results emphasise the interesting nature of immune checkpoint shifting therapies, which have potential clinical applications in the context of colorectal cancer.

## Background

Colorectal cancer (CRC) is the third-most common cancer worldwide and the second in Europe [1–3]. Current treatments include tumour surgery in the early stages, followed by chemo- and radiotherapies for patients in advanced stages [4]. Although the 5-year life expectancy is close to 90% with early detection, up to 40% of patients experience recurrence, mainly in the form of regional or distant metastases [5], which has driven oncologists to search for further preventive treatments such as immunotherapies [6].

Immunotherapy is a rapidly expanding field, and significant effort is being made to improve the antitumour immune response by shifting immune checkpoint (IC) molecules [7, 8]. IC molecules are key regulators of T cell activation and self-tolerance [9], offering a new avenue of potential therapeutic targets in immune response diseases. ICs comprise a heterogeneous superfamily of molecules that either costimulate or inhibit T cell responses to mediate immune tolerance and mitigate collateral tissue damage when the immune system is responding to pathogenic infection [10]. Pathological conditions affecting both the adaptive and the innate systems, such as cancer progression, have been observed to evolve by altering the expression of these proteins. The surface ICs might act as ligands of lymphocyte receptors, modulating the duration and range of the adaptive immune response, as both stimulatory and adaptive response inhibitors. Within the inhibitory ICs, members of the B7 superfamily of molecules, and especially the ligand of programmed cell death proteins (PD-L1), emerge as promising molecules in various clinical contexts [11–13].

Unfortunately, there are still no effective immunotherapies for CRC [14]. The continuous interaction of the gastrointestinal tract with pathogens, and hence the continuous action of the immune system on this tissue, represents a problem for the use of immune-based therapies [15, 16]. Nevertheless, initial studies have shown the importance of the immune system in prognoses, highlighting the crucial role of tumour-infiltrating cells such as lymphocytes, natural killer cells and macrophages [17]. In addition, analyses of the type, density and location of tumour-infiltrating immune cells within CRC tumour samples have revealed that, in addition to genetic mutations and tumour/node/metastasis staging, immunological data are an independent predictor of patient survival [18, 19]. Along these lines, there is broad consensus in that the subset of patients with a clinical response to PD-1 therapy consist of those harbouring a tumour microsatellite instability-high (MSI-H) phenotype, also called deficient DNA mismatch repair (dMMR) CRC, in contrast to proficient mismatch repair (pMMR) CRC [20], with MLH1, MSH2, MSH6 and PMS2 as the main MMR gene products.

Herein, we have analysed the presence and primary features of monocytes and T-lymphocytes in both tumour and peritumour tissues of patients with CRC, to elucidate which are the main tumour cells involved with ICs effects. Our data reinforce the importance of innate immune cells in the tumour microenvironment context, emphasising that crosstalk between tumour cells and immune components is significantly driven through the interaction of PD-L1/PD-1 immune checkpoint shifting, despite pMMR status.

## Methods

### Study design

Fifteen patients fulfilling the diagnostic criteria for colon adenocarcinoma resection were surgically treated. A radical right colectomy with ileotransversostomy anastomosis was performed on all of them. Samples from the tumours and their surrounding (peritumour) areas were collected during the surgery. Histological diagnoses were based on microscopic features of the carcinoma cells, thus determining the histological type and grade. The clinical data on the patients included in the study are summarised in Table 1. All the patients provided informed consent to participate in the study, which was approved by the Institutional Review Board of La Paz University Hospital.

**Table 1:** Cohort description

Characteristic	Value	%
All patients (*n* = 15)		
Age, years	73.80 ± 7.79	
Sex		
Male	10	66.7
Female	5	33.3
Tumour location		
Caecum	4	26.7
Ascending colon	8	53.3
Transverse colon	3	20
Emergency surgery		
Yes	2	13.3
No	13	86.7
Surgical procedure		
Laparoscopic right haemicolectomy	13	86.7
Open right haemicolectomy	2	13.3
TNM^a^ stage		
I	1	6.7
IIA	6	40
IIB	3	20
IIIA	0	0
IIIB	2	13.3
IIIC	1	6.7
IVA	2	13.3
IVB	0	0
Adjuvant chemotherapy		
Yes	6	40
No	9	60
Hepatic metastases		
No	12	80
Synchronous metastases	2	13.3
Metachronous metastases	1	6.7
MMR status		
pMMR	13	86.6
dMMR	1	6.7
Unknown	1	6.7

^a^*TNM* tumour-nodes-metastasis classification

### Microsatellites stability analyses

The status of DNA mismatch repair proteins was assessed by performing immunohistochemistry directed against MLH1, PMS2, MSH2 and MSH6, over areas of infiltrative adenocarcinoma previously selected on haematoxylin-eosin slides. Adequate internal and external controls were used in each case. Protein loss was identified by a complete absence of nuclear staining in malignant cells. Tumours with retained expression of the four proteins were considered stable, whereas tumours in which one or more proteins were lost were considered unstable [21]. Whenever the interpretation was doubtful, the results were further analysed by polymerase chain reaction with a commercial kit, as specified by the manufacturer (Promega, MD1641), to compare tumour and nontumour tissue areas. Tumours were considered dMMR when more than two out of the five markers examined were unstable [22]. The results are summarised in Table 1.

### Isolation and culture of cancer cells

We followed standardised protocols [23]. Briefly, fresh tumour and peritumour tissue samples were washed in phosphate-buffered saline (PBS) solution containing a mixture of antibiotics (gentamicin, fungizome/amphotericin-B and penicillin/streptomycin), gently shaking for 15 min at room temperature. Next, samples were chopped into pieces of approximately 1 mm^3^ and enzymatically digested with collagenase-P (1 mg/mL, SIGMA) in PBS, gently shaking for 30 min at 37 °C. After centrifugation, the supernatants were sieved with a 70-μm cell strainer and seeded on nontreated Costar plates: the tumour supernatants were cultured in selective Dulbecco’s Modified Eagle Medium (DMEM)/F12 (enriched media with 5 mM hydroxyethyl piperazineethanesulfonic acid (HEPES), serum-free supplements B-27 (0.2%) and N-2 (1%), 20 ng/mL basic fibroblast growth factor (bFGF) and 10 ng/mL epidermal growth factor (EGF) supplements) and peritumour samples in DMEM, both with 10% foetal bovine serum (FBS) and antibiotics (gentamicin, fungizome and penicillin/streptomycin). All the cell cultures were performed at 37 °C in a 5% CO_2_ humidified incubator. Images were acquired with a Leica CTR6000 microscope. Aliquots were taken immediately after finishing the isolation protocol and markers expression were analysed by fluorescence-activated cell sorting (FACS).

### Reagents

Roswell Park Memorial Institute (RPMI) medium and DMEM (Invitrogen) were used for the cell cultures. The following antibodies were used for the FACS analysis: α-CD14, α-CD4, α-CD8, α-CD3 (Immunostep); α-PanK, α-EpCAM, α-PD-1, α-PD-L1, α-CD163, α-CD133, α-CD64, α-EphBR2, α-vimentin (MiltenyiBiotec); α-CD34 (BD Pharmingen); α-CD90 and α-CD45 (Labclinics eBioscience). The carboxyfluorescein succinimidyl ester (CFSE) for the proliferation assays was purchased from Thermo Fisher. To inhibit PD-L1/PD-1 interaction, an α-PD-1 antibody was used (Bristol-Myers Squibb). All the reagents were endotoxin-free, as assayed with the Limulus amoebocyte lysate test (Cambrex).

### Flow cytometry

For marker staining, the cells were labelled with the specific monoclonal antibodies and incubated for 30 min at 4 °C in the dark. For the unconjugated antibodies, secondary host-matched conjugated antibodies were added and incubated for another 30 min. Matched isotype antibodies were used as negative controls. Data were acquired by flow cytometry using a BD FACSCalibur flow cytometer (BD Biosciences) and analysed with FlowJo vX.0.7 software (FlowJo, LLC).

### Proliferation assays

Due to the availability of tumour cells, seven of the 13 pMMR patients were assessed for proliferative capacity. Peripheral blood mononuclear cells (PBMCs), isolated from two healthy volunteers by standardised protocol [24], were seeded in a 96-well plate (10^5^ per well) in complete RPMI, and cocultured or not (naïve control; φ) with 5 × 10^4^ tumour (T) cells from CRC samples. An α-PD-1 antibody was used to a final concentration of 5 μg/mL to block the PD-L1/PD-1 interaction. Thereafter, we stained the cultures with CFSE-fluorescein isothiocyanate (FITC) following the manufacturers’ instructions, and let them grow for 5 days before measuring CFSE dimming by FACS.

### Statistical analysis

The number of experiments analysed is indicated in each figure. For the analysis, Wilcoxon matched paired tests were used. The statistical significance was set at *p* < .05, and the analyses were conducted using Prism 5.0 software (GraphPad).

## Results

### Isolated tumour cells show an activated stemness-like phenotype

**Fig. 1:**
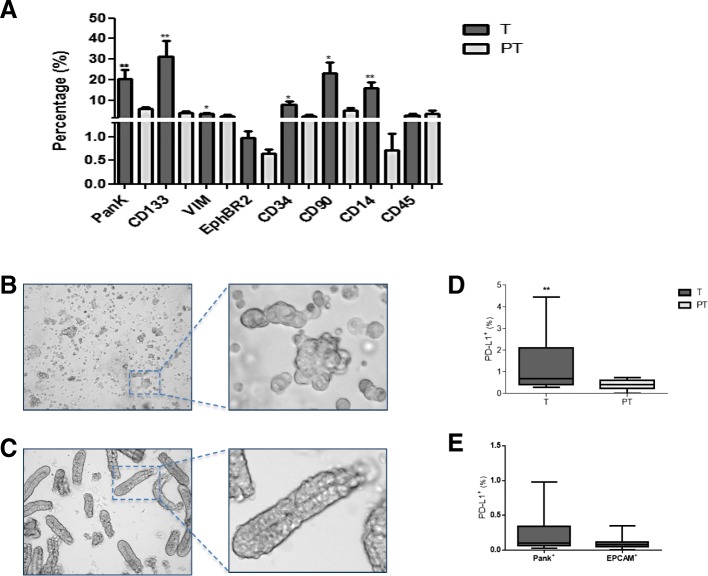
Phenotypic characterisation of isolated cells from colorectal cancer samples. Percentage of surface marker expression in cells from tumour (T, grey) vs. peritumour (PT, white) areas, immediately after isolation from patients with CRC (*n* = 15), as determined by FACS (**a**). Morphological appearance (10×) of tumour (**b**) and peritumour cells (**c**) immediately after isolation procedure. Percentage of PD-L1^+^ cells within tumour (T, grey boxes) and peritumour (PT, white boxes) areas, as measured by FACS (**d**). Percentage of PD-L1^+^ cells on PanK^+^ and EpCAM^+^ gated tumour cells from D (**e**). * *p* < .05, ** *p* < .01 using a Wilcoxon test

Phenotyping of cells isolated from tumour and peritumour samples from patients with CRC (Fig. 1) revealed significantly different profiles (Fig. 1a). The former had a higher expression of some colorectal cancer (PanK, CD133), mesenchymal (vimentin), stemness (CD34, CD90) and immune system (CD14) markers, as expected from activated tumour cells. After isolation, tumour cells appeared with morphological features resembling stem cell-like spheroids and aggregates (Fig. 1b), unlike the PT cells (Fig. 1c). Moreover, cells from the tumour area showed limited expression of the immune-checkpoint molecule PD-L1, but significantly greater expression than those cells isolated from the peritumour region (Fig. 1d). However, the percentages of PD-L1^+^ cells were quite low in both PanK and EpCAM (*epithelial cell adhesion molecule*)-positive subpopulations (Fig. 1e).

### Tumour but not peritumour areas are enriched in immune populations

**Fig. 2:**
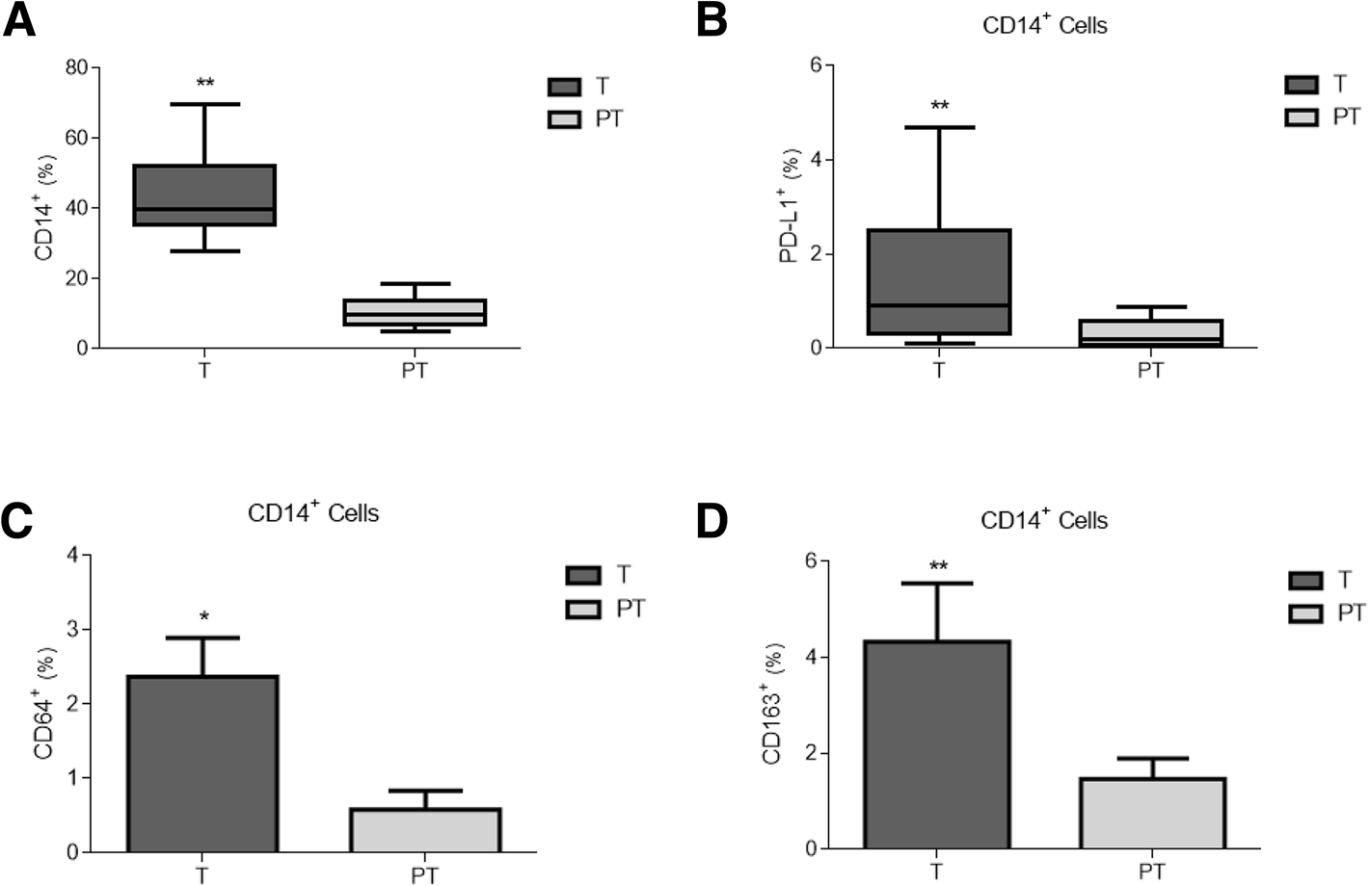
Phenotype of infiltrated monocytes/macrophages in tumour and peritumour areas in colorectal cancer samples. Percentage of infiltrated CD14^+^ cells within tumour (T, grey boxes) and peritumour (PT, white boxes) areas, as measured by FACS (**a**). Percentages of PD- L1^+^ (**b**), CD64^+^ (**c**) and CD163^+^ (**d**) cells on CD14^+^ gated populations in C. * *p* < .05, ** *p* < .01 using a Wilcoxon test

We next characterised the immune populations infiltrated in both the tumour and peritumour tissues. As shown in Fig. 2a, there was patent monocyte infiltration of the tumour, but not in the surrounding tissue. Curiously, tumour-infiltrated monocytes/macrophages expressed higher levels of PD-L1 than those in the surrounding area (Fig. 2b). As expected [25], CD14^+^ cells in tumour tissue exhibited an M2-like alternative phenotype, as shown by their higher expression of CD64 (Fig. 2c) and CD163 (Fig. 2d) with respect to peritumours.

**Fig. 3:**
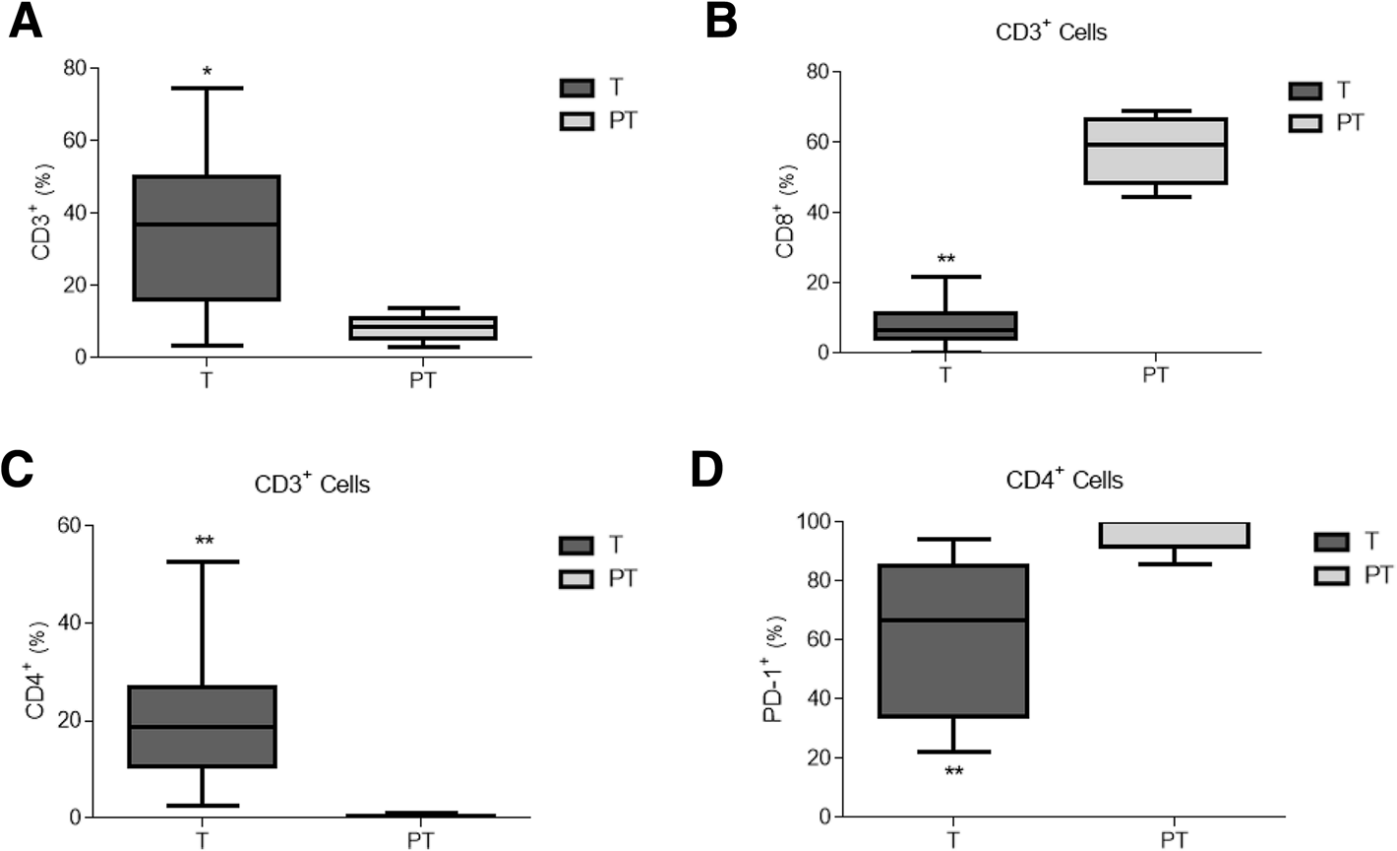
Characterisation of infiltrated T-lymphocytes in tumour and peritumour areas in colorectal cancer samples. Percentage of CD3^+^ cells within tumour (T, grey boxes) and peritumour (PT, white boxes) areas from patients with CRC (*n* = 15), as analysed by FACS (**a**). Percentage of CD8^+^ cells (**b**) and CD4^+^ cells (**c**) on CD3^+^ gated populations in A. Expression of PD-1 on CD4^+^ gated populations in C (**d**). * *p* < .05, ** *p* < .01 using a Wilcoxon test

In terms of the study of the lymphoid cell lineage, we found a large number of CD3^+^ cells in the tumour, but not in the peritumour samples (Fig. 3a). Moreover, the peritumour CD3^+^ population was enriched in CD8^+^ cells (Fig. 3b), whereas CD4^+^ were found in the majority of CD3^+^ tumour cells (Fig. 3c). Interestingly, most of these CD4^+^ lymphocytes were also PD-1^+^ in both areas studied (Fig. 3d).

### PD-L1/PD1 crosstalk controls T-cell proliferation

**Fig. 4:**
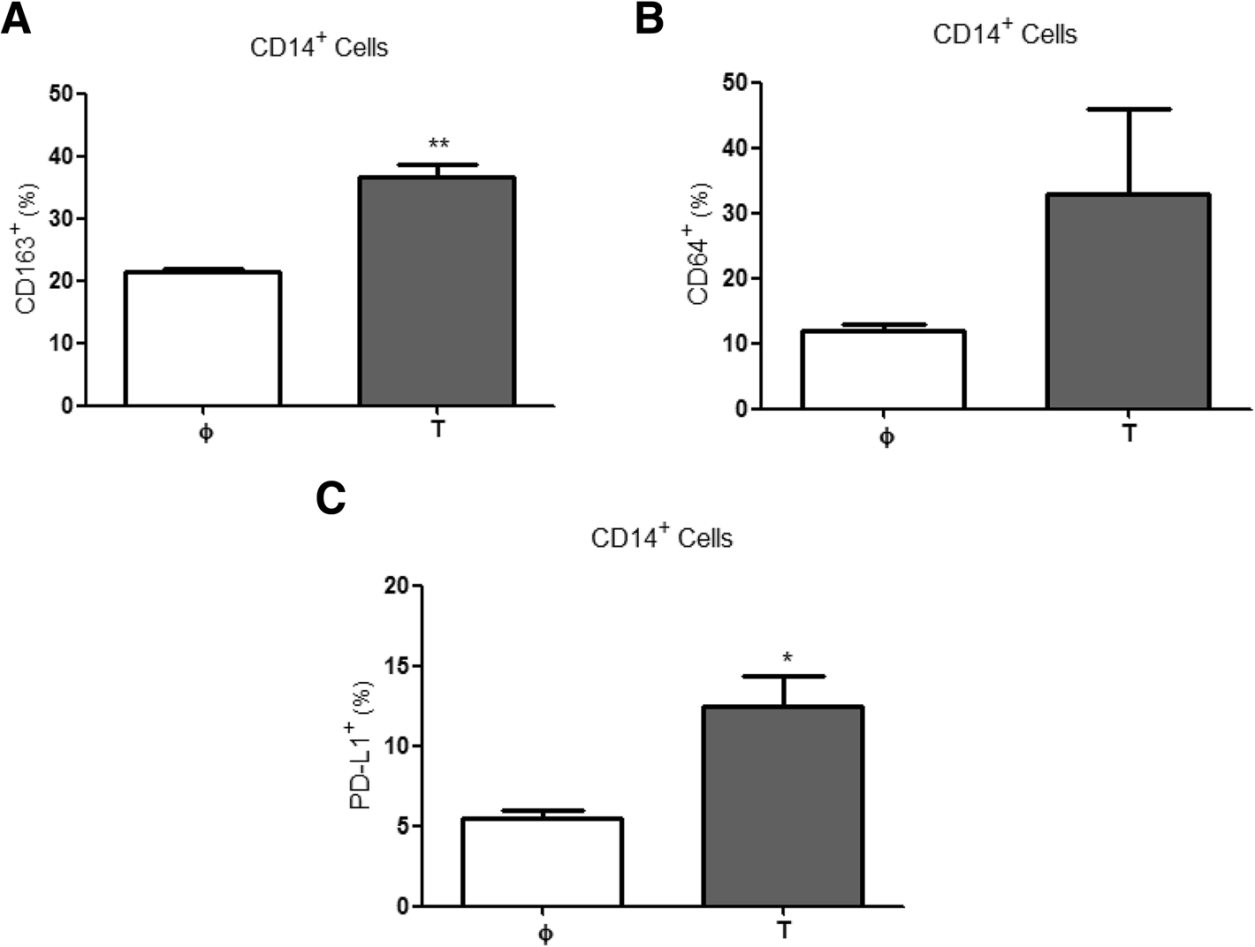
Monocyte/macrophage phenotype in cocultures with colorectal cancer stem cells and lymphocytes. Expression of CD163 (**a**), CD64 (**b**) and PD-L1 (**c**) in CD14^+^ naïve monocytes/macrophages (φ, white boxes) vs. monocytes/macrophages cocultured (T, grey boxes) for 5 days with isolated tumour cells from colorectal cancer tumour samples and lymphocytes. * *p* < .05, ** *p* < .01 using a Wilcoxon test

**Fig. 5:**
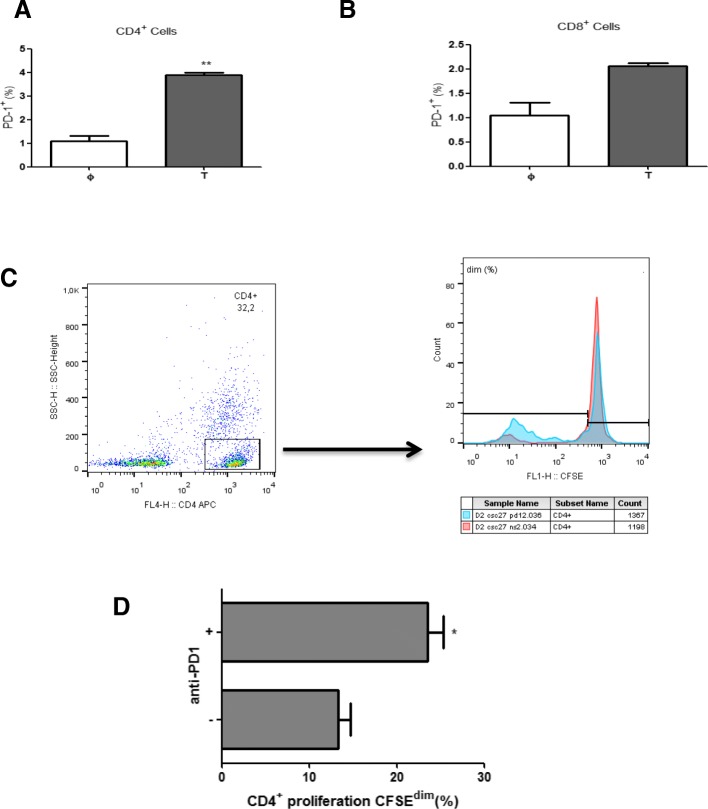
Proliferative properties of T-lymphocytes cocultured with monocytes/macrophages and colorectal cancer stem cells. Expression of PD-1 by CD4^+^ (**a**) and CD8^+^ (**b**) populations within naïve lymphocytes (φ, white boxes) vs. lymphocytes cocultured (T, grey boxes) for 5 days with isolated tumour cells from colorectal tumour samples and monocytes/macrophages. Representative gating strategy to analyse the proliferation of CD4^+^ T-lymphocytes (**c**) and their proliferation in the presence (+) or not (−) of an α-PD-1 antibody, as measured by CFSE dimming (d). * *p* < .05, ** *p* < .01 using a Wilcoxon test

Finally, we explored the crosstalk among the various immune lineages and the pMMR tumour cells in coculture conditions. In this context, after 120 h of interaction, CD14^+^ monocytes/macrophages showed an alternative phenotype (high expression of CD64 and CD163; Fig. 4a, b) as well as higher levels of PD-L1 than naïve controls (Fig. 4c), which did not have any contact with tumour cells. We also noticed that CD4^+^ (Fig. 5a), but not CD8^+^ (Fig. 5b), lymphocytes increased their expression of surface PD-1. Furthermore, CD4^+^ T-lymphocytes from five of these pMMR tumours significantly increased their proliferative capacity when an α-PD-1 antibody was added to the coculture (Fig. 5c, d).

## Discussion

Although cells isolated from CRC patients’ tumours exhibited proper stem cell marker expression, which differs from those with peritumour origin, we did not detect a significantly associated expression of PD-L1. Hence, these data suggest that tumour cells, per se, are not the primary source of PD-L1 in tumour samples. This suggestion is in line with other authors’ reports showing that methylation of the *PD-L1* gene in CRC cells can result in low transcription and translation of PD-L1 in these cells [26].

Due to the increased relevance of immune system components in the tumour microenvironment [27], we first studied the presence and phenotype of infiltrated CD14^+^ monocytes/macrophages, and CD4^+^ or CD8^+^ T-lymphocytes, noting that the tumour zones were significantly enriched in CD14^+^ cells. Our results suggest that not only lymphocytes but also monocytes/macrophages play an important role in the evolution of CRC. These cells serve as the first line of host defence and are equipped to recognise and respond to tumour cells by triggering inflammation. However, we confirmed that tumour-infiltrating monocytes/macrophages exhibited an alternative, M2-like activation [28] in the samples. Thus, their protective influence might be suppressed in this context, given that tumours counteract the cytotoxic and proinflammatory activities (M1 phenotype) of tumour-associated macrophages (TAMs) in their local environments by neutralising them via multiple mechanisms, such as the production of certain tumour cytokines and prostaglandins [29]. In fact, findings on the importance of this polarisation process have not been only reported for CRC progression [30] but also for other several cancers, such as glioblastoma [31], as a consequence of M2-macrophage production of important factors that augment tumour growth (e.g., IL-10).

Furthermore, these infiltrated CD14^+^ cells exhibited high PD-L1 expression, which allowed them to interact with CD3^+^PD-1^+^ T-cells, and might provoke the phenomenon known as ‘T-cell exhaustion’ thus impairing the T-cell response to tumour expansion. PD-L1 expression in CRC cells appears to be paradoxically associated with a high number of CD8^+^ cells [32], and it correlates with early tumour stages. Nevertheless, we found no significant PD-L1 levels on cancer cells or CD3^+^CD8^+^ cells in our tumour samples. In fact, our data showed a significant number of infiltrated CD4^+^ T-cells expressing PD-1, which strongly suggests an interaction with the CD14^+^PD-L1^+^ resident population. These findings are in line with those of Llosa et al. [33], who postulated that CD4^+^ T-cells infiltrated populations, in both pMMR and dMMR CRC tumours, might play an important role in PD-L1/PD-1 axis function through the T-cell exhaustion phenomenon. In addition, we have recently reported a similar behaviour in some other clinical contexts, such as sepsis [34] and obstructive sleep apnoea [35], leading to T-cell exhaustion and the progression of illness due to the inability of T-cells to work steadily.

It is noteworthy that when an α-PD-1 was added to cocultures of naïve immune lineages and pMMR tumour cells, an increase in CD4^+^ cell proliferation was observed, suggesting that the T-cell exhaustion mechanism due to PD-L1/PD-1 crosstalk had been abolished [36, 37]. These data are in agreement with the observed positive outcome of patients with CRC who were treated with new α-PD-1 drugs such as pembrolizumab, nivolumab and other related antagonists [38], and match with the observed predisposition of tumour-infiltrating lymphocytes (TILs) and TAMs in dMMR tumours to respond to immune checkpoint blockade therapy (ICBT) [33, 39]. The benefits of α-PD-1 antibody therapy for pMMR and dMMR tumours remain controversial [40]. Whilst a majority of authors concur on defining dMMR as the sole tumour type able to respond to this therapy [41], there are also various studies stating its relevance against pMMR tumour progression [32, 42]. In this sense, the most common argument for dMMR performance is based precisely on immune-infiltrated population activity in highly mutated MMR-deficiency tumour microenvironment (TME) [43]. However, it could apply also to pMMR (although to a lesser extent of specific TME cases due to their associated gene stability) in which up-regulators of PD-L1 expression (e.g., IFNγ) are present in higher proportions [41]. In addition, the responsiveness to α-PD-1 observed in these pMMR tumours might be linked to alternative mechanisms rather than to a deficiency of DNA repairing enzymes. For all these cases, other features should also be taken into consideration when mechanisms for PD-L1/PD-1 crosstalk are dug into. These features include the generation of neo-antigens that then activate T-cell response to tumours, changes in signalling pathways of chemokines and cytokines expression, and the loss of MHC class I molecules in tumour cells [44]; all indicating the solid implication of TILs and TAMs. Hence, our results reinforce the suitability of either active or passive therapies [43] to restore patient immunity, e.g., M1-macrophage polarisation, together with ICBT.

Finally, some recent studies have found significant differences in PD-L1/PD-1 axis regulation among CRC primary and metastatic lesions. In fact, epigenetic mechanisms, PD-L1/PD-1 regulatory factors in the TME and TILs/TAMs composition, might be quite different in both tissues [41], making regimens for these metastatic patients clinically challenging with a single approach. Even though these differences need further investigation, combinatory treatments for fighting the complex relationships between immune surveillance and the evolving properties in tumour cells appear to be the most advantageous for treatment of the various CRC subtypes. Accordingly, our findings open the possibility of reconsidering combination therapies for pMMR tumours, including both ICBT and driving local infiltrated immune populations into a “naïve-like” stage to overcome tumour resistance.

## Conclusion

Altogether, our results support previous findings indicating that the tumour microenvironment induces the expression of PD-L1 in CD14^+^-TAMs, and that their interaction with CD4^+^PD-1^+^ cells triggers T-cell exhaustion, thus allowing tumour propagation. In this sense, inhibition of the crosstalk at this immune checkpoint, PD-1/PD-L1, would abrogate this effect even in pMMR tumours under specific conditions. Although currently there are still no effective immune therapies for CRC, our results indicate that this line of research should not be abandoned, given both ICs and “naïve-like” stage monocytes/macrophages appear to be crucial for the development of CRC. Therefore, new strategies to avoid such interference must be addressed in this clinical context.

## Abbreviations

CRC: colorectal cancer
DMEM/F12: Dulbecco Modified Eagle Medium/Nutrient Mixture F12 media
dMMR: deficient mismatch repair
IC: immune checkpoint
ICBT: immune checkpoint blocking therapy
PanK: Pan-cytokeratin (Miltenyi, Cat. 130–080-101)
pMMR: proficient mismatch repair
RPMI: Roswell Park Memorial Institute media
TAMs: tumour-associated macrophages
TILs: tumour-infiltrated lymphocytes
TME: tumour microenvironment
